# Increased Accumulation of Medium-Chain Fatty Acids by Dynamic Degradation of Long-Chain Fatty Acids in *Mucor circinelloides*

**DOI:** 10.3390/genes11080890

**Published:** 2020-08-05

**Authors:** Syed Ammar Hussain, Alexis Garcia, Md. Ahsanul Kabir Khan, Shaista Nosheen, Yao Zhang, Mattheos A. G. Koffas, Victoriano Garre, Soo Chan Lee, Yuanda Song

**Affiliations:** 1 Colin Ratledge Center for Microbial Lipids, School of Agriculture Engineering and Food Science, Shandong University of Technology, Zibo 255049, China; ammarshah88@yahoo.com (S.A.H.); akabir08@gmail.com (M.A.K.K.); shaista_nosheen@yahoo.com (S.N.); zhangyao@sdut.edu.cn (Y.Z.); 2 Department of Biology, South Texas Center of Emerging Infectious Diseases (STCEID), University of Texas, San Antonio, TX 78249, USA; alexis.garcia2@utsa.edu; 3 Department of Chemical and Biological Engineering, Center for Biotechnology and Interdisciplinary Studies, Rensselaer Polytechnic Institute, Troy, NY 12180, USA; koffam@rpi.edu; 4 Departamento de Genética y Microbiología (Unidadasociada al IQFR-CSIC), Facultad de Biología, Universidad de Murcia, 30071 Murcia, Spain; vgarre@um.es

**Keywords:** *Mucor circinelloides*, metabolic engineering, acyl-ACP-Thioesterase, acyl-CoA-oxidase, acyl-CoA-thioesterase, β-oxidation pathway, green biofuels, functional lipids

## Abstract

Concerns about global warming, fossil-fuel depletion, food security, and human health have promoted metabolic engineers to develop tools/strategies to overproduce microbial functional oils directly from renewable resources. Medium-chain fatty acids (MCFAs, C8–C12) have been shown to be important sources due to their diverse biotechnological importance, providing benefits ranging from functional lipids to uses in bio-fuel production. However, oleaginous microbes do not carry native pathways for the production of MCFAs, and therefore, diverse approaches have been adapted to compensate for the requirements of industrial demand. *Mucor circinelloides* is a promising organism for lipid production (15–36% cell dry weight; CDW) and the investigation of mechanisms of lipid accumulation; however, it mostly produces long-chain fatty acids (LCFAs). To address this challenge, we genetically modified strain *M. circinelloides* MU758, first by integrating heterologous acyl-ACP thioesterase (*TE*) into fatty acid synthase (FAS) complex and subsequently by modifying the β-oxidation pathway by disrupting the acyl-CoA oxidase (*ACOX*) and/or acyl-CoA thioesterase (*ACOT*) genes with a preference for medium-chain acyl-CoAs, to elevate the yield of MCFAs. The resultant mutant strains (M-1, M-2, and M-3, respectively) showed a significant increase in lipid production in comparison to the wild-type strain (WT). MCFAs in M-1 (47.45%) was sharply increased compared to the wild type strain (2.25%), and it was further increased in M-2 (60.09%) suggesting a negative role of *ACOX* in MCFAs production. However, MCFAs in M-3 were much decreased compared to M-1,suggesting a positive role of *ACOT* in MCFAs production. The M-2 strain showed maximum lipid productivity (~1800 milligram per liter per day or mg/L.d) and MCFAs productivity (~1100 mg/L.d). Taken together, this study elaborates on how the combination of two multidimensional approaches, *TE* gene over-expression and modification of the β-oxidation pathway via substantial knockout of specific *ACOX* gene, significantly increased the production of MCFAs. This synergistic approach ultimately offers a novel opportunity for synthetic/industrial biologists to increase the content of MCFAs.

## 1. Introduction

Over the last two decades, microbially derived oils have offered a platform to face the challenges related to global warming, scarcity of non-renewable recourses, and food security [1,2,3]. Biokerosene is fundamentally composed of medium-chain-length alkanes that could be easily derived from saturated fatty acids with medium-chain length (MCFAs) [2]. In the current scenario of lowering environmental impact of fossil-fuel, aviation industry is paying a great attention to the development of green-fuels. These fuels have a number of necessities: they must exist as liquid at low temperature and have high energy content by volume. These MCFAs also play a significant role as promising intermediates for the diverse nutraceutical, biochemical, and chemical industries, such as the manufacturing of surfactants, detergents, bio-plasticizers, adhesives, lubricants, perfumes, and precursors for flavor and floral aromas. Furthermore, MCFAs have also been gaining attention in the context of a balanced diet and human health concerns because of their ease of absorption, robust translocation to the liver through the portal veins, and swift metabolism via the β-oxidation pathway, ultimately enhancing diet-triggered thermogenesis. This mechanism has prompted interest in their use for the prevention and management of different metabolic disorders, such as hypertension, atherosclerosis, hyperlipidemia, type-II diabetes, obesity, and cardiovascular diseases (CVDs). Moreover, the abundance of naturally produced MCFAs from plant sources (i.e., mostly from palm and coconut) has been anticipated to be insufficient to circumvent industrial demand. Thus, the recent focus of investigation has been diverted toward the de novo biosynthesis of MCFAs from diverse oleaginous microorganisms [1,4]. These microorganisms have been genetically manipulated to produce multi-purpose products of biotechnological interest [5,6]. *Mucor circinelloides* Wj11 has an innate ability to accumulate oils up to 36% of the cell dry weight (CDW) [7]. Owing to its high-oil-generating ability, it offers a reliable option for genetic modulation to produce functional lipids and green energy fuels [8,9]. Previously, these fungal strains have been engineered to produce biofuels and microbial lipids consisting mostly of long alkyl-chains (C14 or more carbons), ultimately making them less attractive for diverse industrial applications [8,10,11,12,13,14,15,16,17]. Metabolic engineering of *M. circinelloides* for the production of MCFAs would facilitate the generation of renewable commodities [4,18].

**Figure 1:**
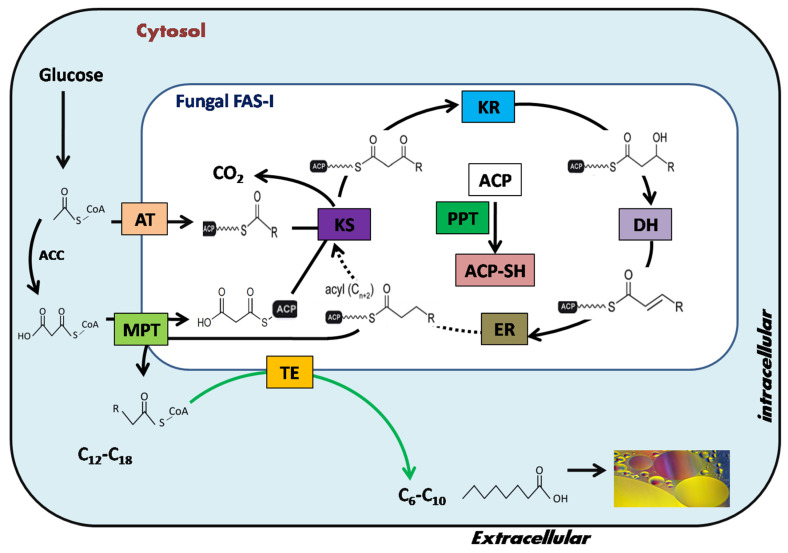
Design and strategy for engineering fungal fatty acid synthase (FAS) in *TE*-over-expressing strains of *Mucor circinelloides* MU758 to overproduce medium-chain fatty acids (MCFAs) contents. Reaction cycles catalyzed by engineered fungal FASs. A heterologous acyl-ACP-thioesterase (*TE*) was integrated into fatty acid synthases (FASs) to release MCFAs (represented by the green arrow). Functional domains are arranged according to reaction sequence. AT: acetyltransferase; KS: ketoacyl synthase; KR: ketoacyl reductase; DH: dehydratase; ER: enoyl reductase; MPT: malonyl/palmitoyltransferase; ACP: acyl carrier protein; PPT: phosphopantetheinyl transferase; ACC: acetyl-CoA carboxylase.

Fatty acids are produced in the cytosol of *M. circinelloides* through fatty acid synthase-I (FAS-I). These FASs enzymes are indispensable to the organism because they are involved in the fatty acid biosynthetic machinery. The detailed mechanism responsible for acyl-chain length elongation and termination has been elaborated in our recent work [4] and by some other groups [19,20]. In brief, no thioesterase (*TE*) activity (the key enzyme governing the fate of fatty acid chain length) was detected in fungal FAS-I (PDB ID: 3HMJ) in comparison to the plant and bacterial counterparts. The acyl-chain elongation is terminated by malonyl/palmitoyltransferase (MPT) rather than TE protein [4,19,20]. The complete mechanism of fatty acid elongation and termination in geneticallymodulated fungal FAS is depicted in Figure 1.

**Figure 2:**
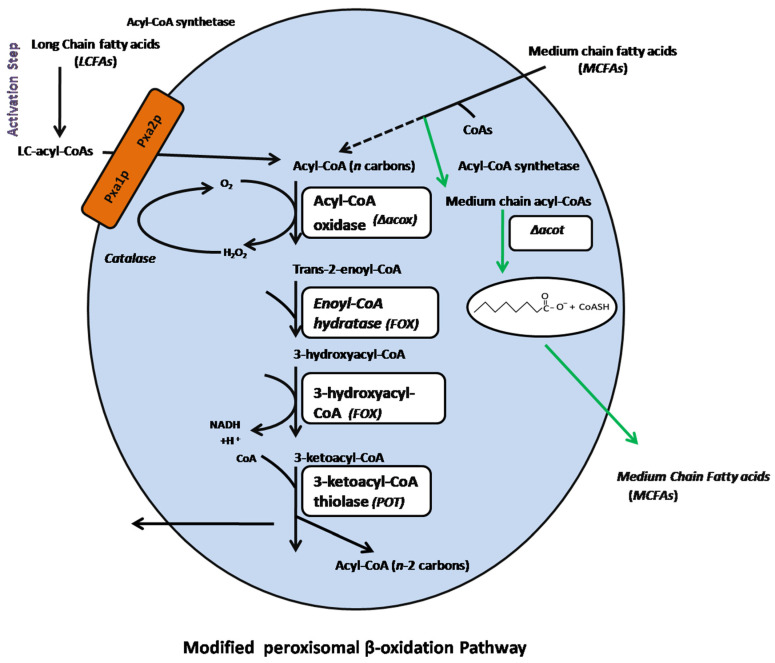
Modification of the β-oxidation pathway in *Mucor circinelloides* strain MU758 (model adapted from Chen et al. [24]). The black lines (solid + dashed) represent the original pathways for long and medium-chain fatty acid degradation steps in peroxisomes. The green line demonstrates the possible route for MCFAs in peroxisomes after disruption of the *ACOX* gene (i.e., exhibiting specificity for medium chain acyl-CoAs) (i.e., M-2). The *ACOT* gene was also disrupted (in the case of M-3), which has a substrate preference for catalyzing the hydrolysis step for acyl-CoA molecules into free fatty acids (FFAs) and coenzyme A (CoAs), eventually facilitating the outward-transportation event in peroxisomes by specific transporters (not shown).

In addition to the distinctive molecular configuration and mechanism of fungal FASs action, the β-oxidation pathway also plays a pivotal role in the plant, fungal and animal kingdoms [21]. In fungi and plants, fatty acid oxidation is executed exclusively in peroxisomes [22]. Peroxisomal β-oxidation enables fungi to degrade long and MCFAs for eventual utilization of acetyl-CoA as the sole carbon and energy source for growth. This pathway is intimately coupled to break down both extracellular and intracellular fatty acids originating from outside source and FAS pathway [23,24]. The detailed mechanism of fatty acid catabolism in the genetically manipulated peroxisome is illustrated in Figure 2. Previously, various approaches have been adopted in different microorganisms to improve the contents of MCFAs, such as engineering to disrupt/reverse the β-oxidation pathway in *Escherichia coli* and *Saccharomyces cerevisiae*; diverse genetic modulation ofmulti-dimensional proteinsrecognized as FASs in *Yarrowia lipolytica*, *S. cerevisiae*, and *E. coli* by modification of the keto-synthase (KS) and acetyl-transferase (AT) binding sites; and tailoring of the magnitude of the KS active pockets and ACP-acyl chain binding sites and incorporating of domain swapping of malonyl-palmitoyl transferase (MPT) with TE protein. Taken together, the aforesaid investigations have reported a low-abundance of MCFAs, and some depend on the addition of specific fatty acids in the culture medium [24,25,26,27,28,29]. Moreover, most oleaginous microorganisms naturally generate mostly long-chain (16 and 18-carbon) fatty acids (LCFAs), which are attributed to the preferential activities of transacylases and acyl-ACP-thioesterases toward a longer acyl-AC chain length [30,31]. Furthermore, these microorganisms have no natural affinity to produce MCFAs due to the absence of MCFAs-producing pathways. However, there is an increasing demand for MCFAs production as a substrate for the functional food and jet-fuel industries, due to their unique physiochemical characteristics [3,5,6,18,32,33,34,35,36,37,38,39,40,41,42,43,44]. To address the above-discussed challenges, there is an immediate need for the selection of an appropriate oleaginous microorganism and suitable strategy/tool. Therefore, in the current investigation, we employed a novel synergistic approach to overproduce MCFAs contents. To achieve our aim, an optimized lipid-tailoring heterologous TE protein from our recent investigation (M65 is uracil auxotroph of *M. circienlloides* Wj11) [4] was first integrated into strain MU758 (uracil and leucine auxotroph strain) into native fungal FAS, followed by modification of the peroxisomal β-oxidation pathway by disruption of the gene encoding acyl-CoA oxidase (*ACOX*) protein with a substrate preference for medium-chain acyl-CoAs. The latter approach was adopted to sustain the oxidation of LCFAs and to avert the oxidation of MCFAs (Figure 2). We screened high lipid producer mutants that demonstrated the required fatty acid portfolio (i.e., C8 to C12). Furthermore, the *ACOT* gene, exhibiting a substrate preference for the hydrolysis of medium-chain CoAs, was also disrupted to verify its involvement in fatty acid breakdown into free fatty acids, as well as the transportation of MCFAs out of peroxisomes. This combinatorial methodology proves an excellent opportunity to produce MCFAs in high abundance.

## 2. Material and Methods

### 2.1. Strains, Plasmids, Cultivation, and Transformation Conditions

The strains and plasmids used in the current investigation are listed in Table 1. For sporulation, *M. circinelloides* strain MU758 (i.e., the uracil and leucine auxotroph strain MU758, which has comparatively similar oil-producing/physiological characteristics to *M. circinelloides* WJ11 [7], was allowed to grow on YPG medium (yeast peptone glucose agar: 3 g/L yeast extract, 20 g/L glucose, 10 g/L peptone, 2% agar, pH 4.5) at 26 °C in the light for 3 d. The medium was supplemented with 200 μg/mL uridine as needed. Subsequently, for spore collection, 8.5% NaCl solution was added to each Petri plate, and the spores were gently scraped with a glass spreader. For the transformation experiments, spore counting was carried out with a hemocytometer. Transformation was carried out using an electroporation-mediated procedure as previously described by Lee et al. [45]. Minimal medium with casamino acids (MMC) (pH 3.2) containing 0.5 M sorbitol was used for transformation experiments [4,46,47,48,49,50]. *M. circinelloide*s pores are multinucleate in nature. Thus, to segregate the homokaryotic transformants, the vegetative selection protocol was carried out by growing the heterokaryotic transformants on MMC (pH 4.5) medium to produce homokaryotic transformants. To select mutants that impulsively eliminate the *pyrF* marker gene (encodes for uracil biosynthesis), 5-fluoroorotic acid (2.5 mg/mL) was added to the YPG medium [49]. All the mutant (i.e., Mu-TE+, the strain harboring the TE-over-expressing plasmid, hereafter referred to as M-1; Mu-TE+, [*acoxΔ*], the strain harboring the TE-over-expressing plasmid and exhibiting the disrupted allele for the *ACOX* gene, hereafter referred to as M-2; and Mu-TE+ [*acoxΔ*, *acotΔ*], the strain harboring the TE-over-expressing plasmid and exhibiting the disrupted allele for *ACOX* and *ACOT* genes, hereafter referred to as M-3) and the wild-type (used as the control, hereafter referred to as WT) strains of *M. circinelloides* (MU758) were initially cultivated by using 80 μL spore suspension (~10^7^ spores/mL) in 1.0-L flasks containing 150 mL of Kendrick and Ratledge medium (K and R); 30 g/L glucose, 3.3 g/L diammonium tartrate, 7.0 g/L KH_2_PO_4_, 2.0 g/L Na_2_HPO_4_, 1.5 g/L MgSO_4_·7H_2_O, 1.5 g/L yeast extract, 0.1 mg/L CaC1_2_·2H_2_O, 8 mg/L FeC1_3_·6H_2_O, 1 mg/L ZnSO_4_·7H_2_O, 0.1 mg/L CuSO_4_·5H_2_O, 0.1 mg/L CO (NO_3_)·6H_2_O and 0.1 mg/L MnSO_4_·5H_2_O, pH 6.0) [4,9,16,17,51,52,53]. Subsequently, these flasks were equipped with baffles to improve aeration and finally placed in a shaker at 150 rpm at a temperature of 26 °C for 24 h. the resultant seed culture was used for inoculation at 10% (*v*/*v*) into a 5-L fermenter (BioFlo/CelliGen 120, New Brunswick Scientific, Edison, NJ, USA) containing 1.5 L of modified K and R medium (i.e., 80.0 g glucose/L plus inorganic salts, 2.0 g diammonium tartrate). The fermenters were adjusted at 26 °C and stirred at 700 rpm with aeration at 0.5 vvm. The pH of the culture medium was constantly adjusted to 6.0 by means of auto-control addition of 2.0 M H_2_SO_4_/2.0 M NaOH solutions. Culture samples of mutant and control strains were collected for a pre-specified duration for further analysis (i.e., 12, 24, 36, 48, 60, 72, 84, 96 h) based on the of lipid accumulation characteristics [4,54].

### 2.2. Construction of the Thioesterase (TE) Over-Expressing Plasmid

**Figure 3:**
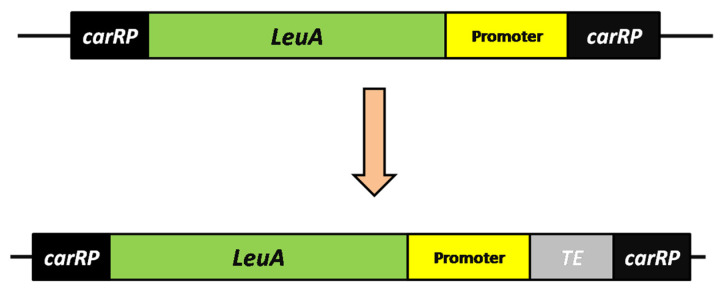
Expression of the *TE* gene. The structure of plasmid pMAT1552-*LeuA*-TE for *TE* gene over-expression in *M. circinelloides* (MU758) is shown. The grey box indicates the coding region of the acyl-ACP-thioesterase *(TE)* gene, while the green rectangle of *LeuA* gene indicates a selectable marker, and the flanking sequences correspond to regions surrounding the carotenogenic *carRP* gene (black boxes) to allow its chromosomal integration by homologous recombination.

The acyl-ACP thioesterase (*TE*) gene was cloned into vector pMAT1552-*LeuA* [4,9] for expression in the *M*. *circinelloides* strain (MU758).The aforesaid vector carried the *LeuA* gene from *M*. *circinelloides* flanked by approximately 1.0 kb of the up- and downstream sequences of the *carRP-carRP* locus (Figure 3). This plasmid was used for the construction of the TE-over-expression vector, as previously described by Zhang et al. [9]. In brief, this vector contains a strong promoter (i.e., *Pgpd-1* (accession No.: AJ305345), the *LeuA* gene (encodes for leucine biosynthesis) as a selectable marker; the surrounding sequences analogous to the flanking sequence of the carotenogenic gene (i.e., *carRP-carRP* locus) facilitate chromosomal integration via homologous recombination (HR). The *TE* gene was amplified from the genome of *Umbellularia californica* (GenBank accession No.: KR180394) by PCR with specific primer pairs (Supplementary Materials: Table S1). The primers contained sequences (30 bp) flanking the restriction site *XhoI.* The resulting PCR fragment was finally cloned into an expression vector (i.e., pMAT1552-*LeuA*). The restriction endonucleases enzyme *XhoI* was to generate the mutant plasmid designated as pMAT1552-*LeuA*-TE (One-Step Cloning Kit from Takara Bio USA, Inc., Mountain View, CA, USA). All primers used for plasmid construction and gene conformation are listed in Supplementary Table S1.

### 2.3. Construction of a Green pyrF Marker

**Figure 4:**
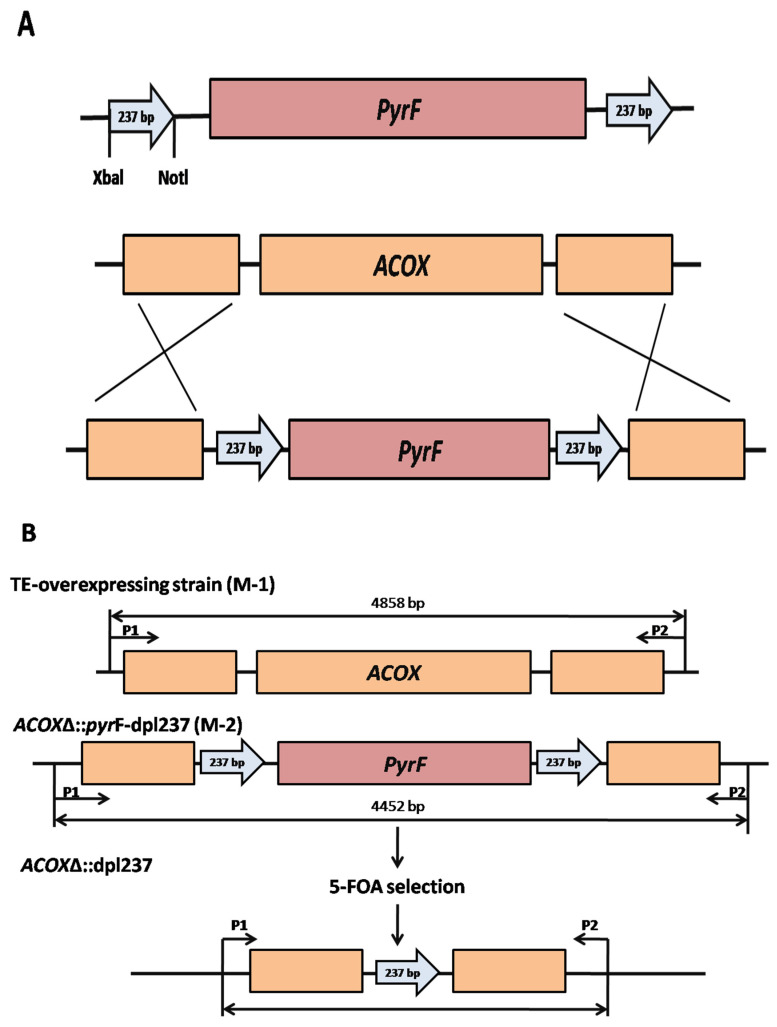
Disruption of the *ACOX* gene and confirmation of the excision of the *pyrF* marker. (**A**) Homologous replacement of the *ACOX* gene with *the pyrF* recyclable marker. (**B**) Confirmation of the excision of the *pyrF* marker. Strain MYS720 (*acoxΔ*::*pyrF-dpl237*) was inoculated on yeast peptone glucose agar (YPG) medium containing 5-FOA. After 3 d of incubation, spontaneous resistant mutants emerged to form a sector from the colony (data not shown). The 5-FOA resistant mutants underwent a vegetative cycle on YPG medium containing 5-FOA. The obtained spores were subjected to polymerase chain reaction (PCR) analysis and auxotrophy tests. Excision of the *pyrF* marker from the *acoxΔ*::*pyrF-dpl237* allele was confirmed by PCR (see details in Section 2.5). The strains with the excised *pyrF* marker did not grow on media without leucine. Two independent (*acoxΔ::dpl237*) strains were obtained.

To construct the *pyrF* recyclable marker, the DNA fragment (890 bp) carrying the *pyrF* gene was amplified from the genomic DNA of *M. circinelliodes* (MU758) using a specific primer pair (YS-275 and YS-276). Subsequently, the previously constructed plasmid pSL13 [55] was used for amplification of the 237bp DNA fragment containing overhanging *Xba*I or *Not*I restriction enzyme recognition sites with primers (YS-277 to YS-280) (Supplementary Table S2), which were flanked by overhanging *Xba*I or *Not*I restriction enzyme recognition sites. All three fragments were joined by overlapping PCR (YS-281 to YS-282) to develop the cassette (1910 bp). Finally, the resulting cassette was cloned into the TOPO vector to generate plasmid pYS719 (Table 1), which harbors a DNA cassette with 237 bp direct repeats on both sides of the *pyrF* gene (Figure 4A).

### 2.4. Disruption of the ACOX and ACOT Genes

The *M. circinelloides* strain contains many acyl-CoA oxidase (*ACOX*) and acyl-CoA thioesterase (*ACOT*) genes with diverse substrate preferences [7]. Based on BLAST analysis, we selected the *ACOX* gene (scaffold00025.39), which has a significant resemblance index to the *ACX3* gene (Gene ID: 837140) from *Arabidopsis thaliana* (substrate specificities for medium acid fatty acids) [56] and the *ACOT* gene (scaffold00002.31), which is a homolog of mouse *ACOT5* (Gene ID: 217698) (substrate specificities for medium chain acyl-CoAs) [57]. To knockout the *ACOX* gene, we assembled a knockout (KO) cassette comprising the *pyrF* blaster marker (*pyrF-dpl237*) flanked by ~1 kb of 5′ and 3′ up- and downstream regions of the *ACOX* gene through overlapping PCR. The 5′ and 3′ regions were amplified with the primers (YS-310, YS-311 and YS-312, YS-313, respectively). Genomic DNA of *M. circinelloides* MU758 strain was used as a template. The *pyrF* blaster (containing the *dpl237* on either side) marker was amplified by utilizing the primer (AS-719 and AS-720). To generate a disruption allele, the following three fragments (50 ng of each fragment) were joined by overlapping PCR using the nested forward and nested reverse primers YS-314 and YS-315. The resulting KO cassette was cloned into the TOPO vector by using the Invitrogen kit according to manufacturer’s instructions with slight modifications.

To disrupt the *ACOX* gene, the bacterial strain was cultured overnight at 37 °C at 200 rpm. The plasmid containing the p*ACOX*-KO cassette was extracted using the Zymo Research Miniprep kit (Irvine, CA, USA) according to manufacturer’s guidelines. The restriction enzyme (Biolab-England) was used to generate the linear fragment required for the *ACOX*-KO cassette. The aforesaid cassette (~3 µg) was introduced into the MU758 strain using the electroporation-mediated method as previously described by Lee et al. [58] to eventually obtain the MYS721 and MYS722 mutant strains (Table 1, Figure 4A,B). To evaluate the functionality of the *ACOT* gene, we further proceeded to disrupt the aforesaid gene in the *ACOX* mutant strains (i.e., *acoxΔ*::*dpl*237). To achieve this goal, a PCR product of the *ACOT* disruption cassette was obtained from plasmid p*ACOT*-KO with primers pairs YS-381 and YS-382 using DreamTaq DNA polymerase (Thermo Scientific, Agawam, MA, USA). Subsequently, the *ACOT* disruption cassette was introduced into the strain MYS722 (*acoxΔ*::*dpl*237) (Figure 4) using our previously described method [49,54]. From 24 independent transformations, we screened seven mutants displaying the required phenotype in comparison to the control and wild-type strain. Two independently obtained mutants, MSA321 and MSA322 (*acoxΔ*::*dpl*237 *acotΔ*::*pyr*F) (Table 1), were selected for further analysis.

### 2.5. Excision of the pyrF Gene from the pyrF Blaster Marker 

Spores of strain MYS720 (*acox*::*pyrF-dpl237*) were grown on YPG medium containing 5-FOA (2.5 mg/mL) and uridine to select mutants that still produced positive colonies but required uridine for growth. The candidate mutants were passaged on YPG medium containing uridine and 5-FOA. Excision of the *pyrF* gene was verified by PCR.

### 2.6. Preparation for Genomic DNA and Quantitative Reverse Transcription Polymerase Chain Reaction (qRT-PCR) Analysis

To confirm the genetic modification, all the mutant strains were allowed to grow on liquid K and R medium for 3 d (under light at 28 °C and 150 rpm). Subsequently, the mycelia were collected by passage through suction filtration assembly. The resulting mycelia were washed twice to remove all possible medium contents. Finally, the genomic DNA was extracted using the DNA quick Plant System Kit (Tiangen Biotech Beijing, Co., Ltd., Beijing, China) according to the manufacturer’s instructions. All the genomic manipulation was confirmed using the PCR amplification protocol with the primers mentioned in Supplementary Table S1. For qRT-PCR analysis, all the mutant (i.e., M-1, M-2, and M-3) and control strain (WT) used in current study were grown in a 5.0-L fermenter with modified K and R medium. Subsequently, the mycelia were harvested at the pre-specified time points of 24, 48, and 72 h. For quantitative reverse transcription PCR (qRT-PCR) analysis, the mutant strains (i.e., M-1, M-2, and M-3) and the wild-type strain (WT) were grown in a 5.0-L fermenter with modified K and R medium, and the mycelia was harvested at 24, 48, and 72 h. Total RNA and qRT-PCR analysis were carried out as previously described method by Zhang et al. [9] using primers (Supplementary Materials: Table S3). The transcriptional level of all the targeted genes were normalized to the levels of 18S rRNA mRNA. The results were represented as the comparative expression level, calculated using the 2^−ΔΔCt^ method.

### 2.7. Determination of Cell Dry Weight (CDW) and Lipid Accumulation

The biomass of all strains (i.e., mutants and control) was collected using the vacuum filtration method. The resulting biomass was washed three times with distilled water to remove all medium contents and then frozen at −80 °C for 3.0 h prior to lyophilization. To calculate the cell dry weight (CDW), the gravimetrical method was used. Lipid extraction was executed as previously described by Folch et al. [59]. Fatty acid methyl ester (FAME) preparation and GC/MS conditions were performed as described in our previous investigations [4,9].

Lipid productivity (*P*_Lipid_) and MCFA productivity (*P*_MCFA_) were calculated as follows:

*P*_Lipid_(mg/L day) = (C*_f_* × *CDW_f_*−C*_i_* × *CDW_i_*)/(*T_f_* − *T_i_*) (1)

*P_MCFA_* (mg/L day) = (C*_MCFA_*(mg/mg TL) × *Lipid* (mg/L)/(*T* (day)) (2)

where C*_f_* is the final lipid content (mg/L), C*_i_* is initial lipid content, *T_f_* is the harvesting time (day) and *T_i_* is the cultivation time (day), CDW*_f_* and CDW*_i_* are the initial and final cell dry weight (mg/L), and TL is the total lipid content.

### 2.8. Determination of Glucose and Nitrogen Concentration in the Culture Medium

To measure the glucose concentration in the culture, the Glucose oxidase-Perid-test kit (Shanghai Rongsheng Biotech Co., Ltd., Shanghai, China) was used, while the indophenol method was used according to Chaney and Marbach [60] to calculate the ammonium concentration.

### 2.9. Separation of Lipid Classes

To estimate the quantity of diverse lipid classes in the engineered strains, total lipids (TLs) were fractioned into their primary components as previously described in our recent investigation [4]. Hydrated florisil (7%) acquired by shaking overnight at room temperature was ultimately used as an adsorbent for the chromatography. Total lipids (TLs) extracted from 50 mL of fungal culture were loaded onto the column, and fractions were eluted in the following solvents ratios: 95:5 hexane/ether, 85:15 hexane/ether, 75:25 hexane/ether, 50:50 hexane/ether, 98:2 ether/methanol, and 96:4 ether/acetate to elute off free fatty acids (FFA), triacylglycerides (TAGs), diacylglycerides (DAGs), monoacylglycerides (MAGs), sterols, and steryl esters, respectively, by using the method as described by Carroll [61].

Abundance of lipid in collected eluents was computed by dichromate reduction procedure as described by Freeman and West [62]. In brief, samples were collected and make completely dry before further analysis. Each dried sample was resuspended in 1.0 mL of 0.2% potassium dichromate dissolved in 96% sulfuric acid (H_2_SO_4_) and boiled for 15 min. Samples were subjected to cool down, and a more 1.0 mL of water was added. Samples were again allowed to cool, and their absorbance was measured at 440 nm to detect formation of Cr^3+^.

### 2.10. Statistical Analysis

A statistical analysis of the obtained data was performed using SPSS 16.0 for Windows (SPSS Inc., Chicago, IL, USA). Differences between means were calculated by the Student’s *t* test, and *p* < 0.05 was regarded as significantly different. The mean value and standard error of the mean were computed from the data obtained from three independent experiments.

## 3. Results

### 3.1. Generation of Heterologous Acyl-ACP Thioesterase (TE) Gene-Over-Expressing Strains

The TE-over-expressing strain (i.e., M-1) was generated to evaluate the role of MCFAs production and lipid accumulation. We chose the best heterologous TE protein from the genome of *U. californica* based on our previous study [4] and inserted it into the expression vector pMAT1552-*LeuA*. (Figure 3 and Supplementary Table S1, Figure S1) (see Section 2.2 for details). Finally, lipid overproducing mutant strains (M-1) were selected for further genetic manipulation experiments.

### 3.2. Generation of the pyrF Blaster Marker and Disruption of the Acyl-CoA Oxidase (ACOX) and Acyl-CoA Thioesterase (ACOT) Genes in the TE-Over-Expressing and acoxΔ::dpl237 Mutant Strains

We sequentially proceeded to disrupt the genes encoding the *ACOX* and *ACOT* proteins in the *TE*-over-expressing and *acoxΔ*::*dpl*237 mutant strains to assess the function of the *pyrF* blaster marker. To achieve our aim, we first targeted the gene encoding acyl-CoA oxidase (*ACOX*) protein, which has a preference for the medium-chain acyl-CoA substrate in the *TE*-over-expressing strain (i.e., M-1). Complete loss of the *ACOX* gene resulted in reduced growth in comparison to the control strain, ultimately confirming the negative effect of the genetic modification on fungal cells (i.e., M-2) (Figure 4A). Transformation and positive colony selection were carried out according to the method described by Rodríguez-Frómeta et al. [8]. To obtain stable mutants with enriched recombinant nuclei, the mutant strains were subjected to three rounds of vegetative growth. Finally, we used the stable mutant strain, (i.e., M-2) MYS-719 (*acoxΔ*::*pyrF-dpl237*) to assess the efficiency of the recyclable *pyrF* marker (Figure 4B, Supplementary Figure S2).

To further confirm the disruption of the *ACOX* gene, we grew spores of the mutant strain MYS-719, which contains only the *acoxΔ* allele instead of the wild type allele. Primers YS-351(P1) and YS-352 (P2) (Supplementary Table S1) outside of the KO cassette were employed to verify the genotype of the progeny. A PCR fragment of 4858 bp was produced from the wild type *ACOX* allele, while a 4452 bp fragment was obtained from the *acoxΔ* allele, eventually validating the absence of the wild-type *ACOX* allele in strain MYS-719 (Figure 4B, Supplementary Table S3).

**Figure 5:**
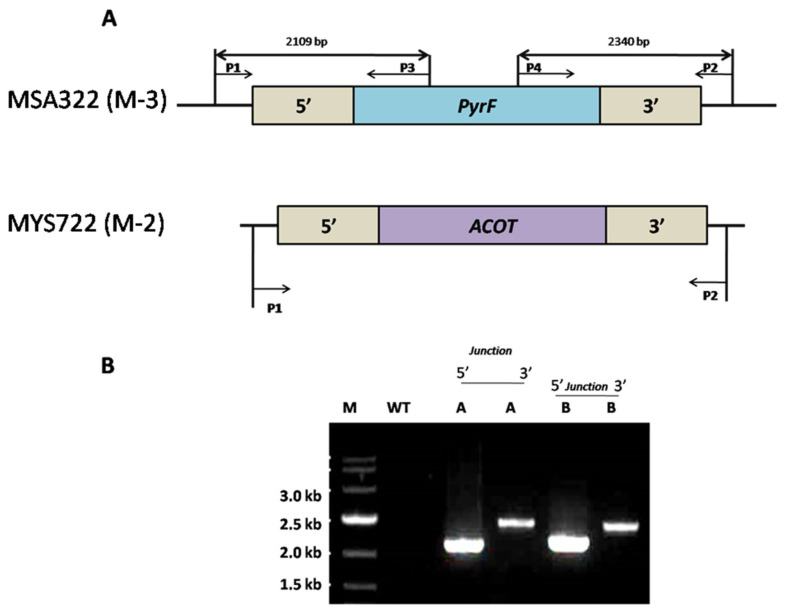
Confirmation of *ACOT* gene knockout in the mutant strains (i.e., M-3); MSA322 (**A**) The *acotΔ*::*pyrF* allele was verified by junction PCR. P1 and P2 primers recognize the sequences outside of the disruption construct. P3 and P4 primers only recognize the *pyrF* gene marker. P1: YS-381; P2, YS-353; P3, YS-354; P4, YS-382 (Supplementary Table S1). (**B**) 5′ and 3′ junction PCR verify the disruption of the *ACOT* gene, where primers P1 and P2 amplify 2109 bp fragment in the mutant strains MYS722 while in the WT the same primers do not amplify the fragment. This outcome confirms the *ACOT* gene was swapped with the *pyrF* gene in the mutant strains, M-3; (MSA322).

Finally, we disrupted the *ACOT* gene (which functions in the hydrolysis of medium-chain acyl-CoAs in peroxisomes) in the strain M-2 background to evaluate the efficiency of the recyclable/green marker and to verify the involvement of *ACOT* protein in medium-chain acyl-CoA breakdown and transportation out of peroxisomes. To achieve this goal, strain MYS-721 (i.e., M-2) was subjected to the transformation procedure to disrupt the *ACOT* gene (Figure 5). Transformation and positive colony selection were carried out according to our previously described method [54]. We screened 21 transformants from 24 independent transformations by PCR amplification using primer pairs YS-381, YS-353, YS-354, and YS-382 (Supplementary Table S1), which underwent three cycles of vegetative passage as previously described for strains M-1 and M-2. The resultant stable strains, MSA321 and MSA322 (i.e., M-3), validated the efficiency of the recyclable *pyrF* blaster marker as well as its utility for a series of gene deletions in *M. circinelloides* MU758 (Supplementary Figure S2A,B). Disruption of the *ACOX* and *ACOT* genes was also verified by qRT-PCR analysis.

### 3.3. Expression Levels of Acyl-ACP-Thioesterase (TE), Acyl-CoA-Oxidase (ACOX), and Acyl-CoA Thioesterase (ACOT) Genes in Mutant Fungal Strains

**Figure 6:**
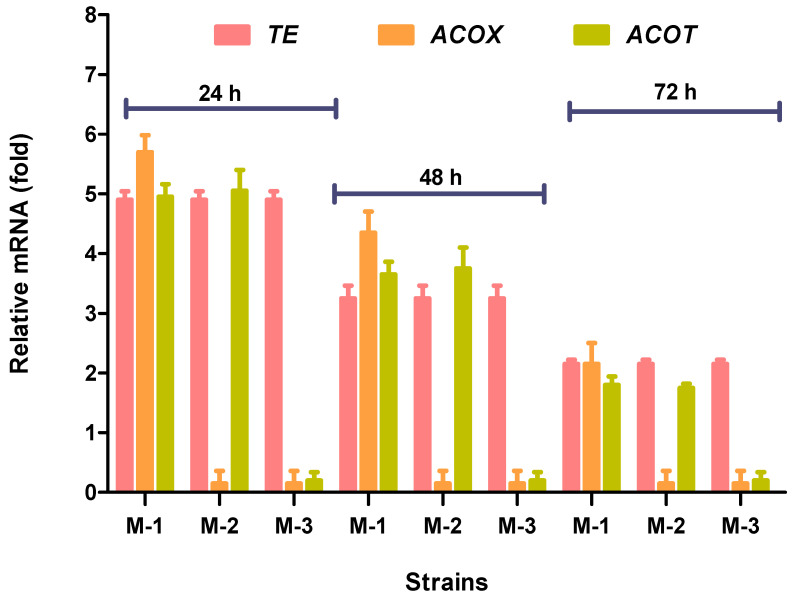
Determination of expression levels of *TE*, *ACOX*, and *ACOT* genes by RT-qPCR in the recombinant strains. Values were the mean of three independent experiments. Error bars represent the standard error of the mean.

Quantitative reverse transcription polymerase chain reaction (qRT-PCR) analysis was conducted to evaluate the mRNA levels of the *TE*, *ACOX*, and *ACOT* genes in the M-1, M-2, and M-3 recombinant strains. All recombinant strains were first grown in a 5-L fermenter with K and R medium and samples were collected at 24, 48, and 72 h growth intervals. The *TE* mRNA was maintained at elevated levels, ultimately confirming that the *TE* gene was truly over-expressed in all mutant strains, (i.e., M-1, M-2, and M-3). In the case of other mutant strains (i.e., M-2, M-3), the M-2 strain was further evaluated for the *ACOX* knockout, while M-3 strain for *ACOX* and *ACOT* knockouts (KOs). *ACOX* and *ACOT* expression were extremely low, and only an insignificant level of mRNA was detected throughout the cultivation period. These results confirmed that the targeted genes had been deleted in these mutant strains (Figure 6).

### 3.4. Fungal Cell Growth and Lipid Yield from Mutant Fungal Strains

**Figure 7:**
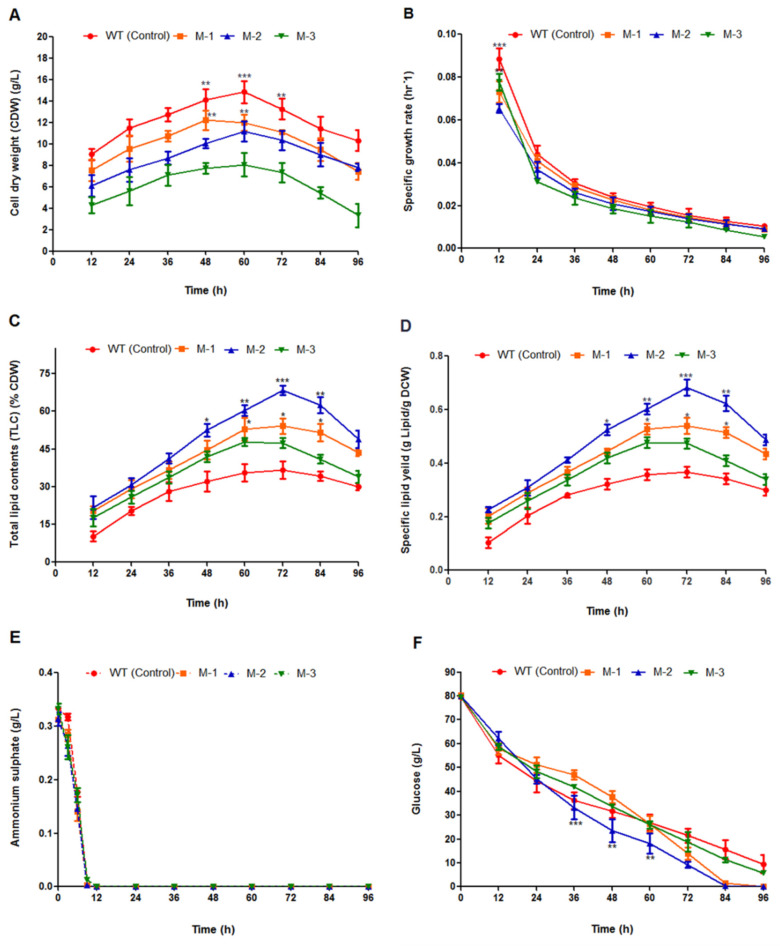
(**A**–**F**) Cell growth, lipid accumulation, and substrate consumption of control (WT) and mutant strains (i.e., M-1, M-2, and M-3) cultivated in a 5-L fermenter with 1.5 L modified K and R medium for 96 h. (**A**) Cell dry weight (CWD), (**B**) specific growth rate, (**C**) percentage of total lipid content from CDW, (**D**) specific lipid yield, (**E**) ammonium sulfate concentration, and (**F**) residual glucose concentration. Values were the mean of three independent experiments. Error bars represent the standard error of the mean. Asterisks indicate that the differences (* *p* < 0.05; ** *p* < 0.01; *** *p* < 0.001) between the means of different treatments are statistically significant.

**Figure 8:**
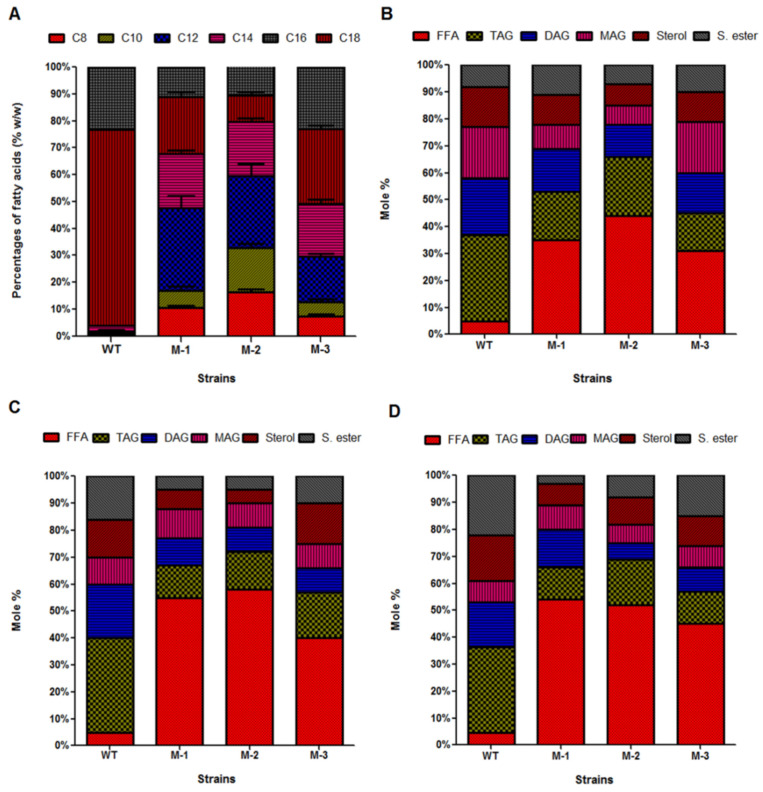
(**A**) Fatty acid (FA) profiles of the control (WT) and engineered strains, (**B**) Abundance of medium-chain lipids, (**C**) 16-carbon lipids, and (**D**) 18-carbon lipids from engineered strains of *M. circinelloides* (MU758) in different lipid classes, i.e., free fatty acids (FFA), triacylglycerol (TAG), diacylglycerol (DAG), monoacylglycerol (MAG), sterol, and steryl ester (S. ester).

Fungal cell growth, total lipid contents, and specific lipid yields in all mutant strains (i.e., M-1, M-2, and M-3) were evaluated in comparison to the control strain (WT). All strains were grown in a 5-L fermenter for 96 h in modified K and R medium (Figure 7A–F). Overall, the growth of all three mutant strains were inhibited (Figure 7A). The CDW of all mutant strains diverged and was lower than the control strain (WT) from the initial cultivation stage (12 h), ultimately demonstrating the effects of the genetic modulation from the onset of cultivation. Moreover, *TE* over-expression and consequent disruption of the *ACOX* and *ACOT* genes in these mutant strains also demonstrated the profound shift in the growth phases, since the majority of the mutant strains, excluding M-3, had a shorter stationary phase. Consequently, growth declined after 72 h of cultivation (Figure 7A,B). The compacted growth has been previously attributed to a shift in the metabolic machinery of the cell towards high lipid production (Figure 7A–D). Similarly, in our case, the genetic manipulation significantly impacted lipid accumulation in strain M-1 and, of note, strain M-2. All engineered strains (i.e., M-1, M-2, and M-3) showed total increases in lipid accumulation ranging from 47 ± 2.4 to 68 ± 2.2%, as compared to 36 ± 2% in the control strain (WT) (Figure 7C). In addition to the total lipid contents (TLC), the engineered strains, i.e., M-1, M-2, and M-3, biosynthesized 0.54 ± 0.013, 0.68 ± 0.041, and 0.47 ± 0.061 g lipid per gram CDW, respectively. In contrast, the control strain (WT) produced 0.36 ± 0.29 g lipid per gram CDW, indicating that the enhanced specific lipid productivity was attributed to the compacted growth of the engineered strains capable of producing non-native fatty acids (Figure 7D). Moreover, the fatty acid portfolio also demonstrated that the genetic manipulation also modified the fatty acid composition in all the mutant strains (Figure 8, Table 2). The nitrogen and glucose consumption rates were comparable among M-1, M-3, and WT, while M-2 strain exhibited comparatively rapid glucose utilization (Figure 7E,F).

*M. circinelloides* strains naturally produced LCFAs (i.e., C16-C18). Combined strategy of *TE* over-expression and the β-oxidation pathway modification by disrupting the *ACOX* gene in M-2 strain played a pivotal role in augmenting MCFAs production. The engineered strains, i.e., M-1 and M-2, generated maximum amounts of total lipids at 72 h of cultivation growth, so we calculated MCFAs abundance at 72 h. Medium chain fatty acids (C8-C12) in the wild type strain were minimum (2.25%), in M-1 were 47.45 ± 1.8%, with the highest being M-2(60.09 ± 2.1%). While the M-3 strain producedcomparatively less MCFAs than M-1 (i.e., 29.61 ± 0.8%) under the same growth conditions. We also speculated that the incremental trend in MCFAs was correlated with the depletion of C18-containing fatty acids (Figure 8A). Fatty acid portfolio of control and mutant strains of *M. circinelloides* MU758 are represented at Table 2.

### 3.5. Incorporation of De Novo Medium-Chain Fatty Acids (MCFAs) into Diverse Lipid Classes

To calculate the potential of mutant strains (i.e., M-1, M-2, and M-3) of *M. circinelloides* MU758 to integrate the de novo MCFAs into different lipid classes, we employed chromatography to fractionate the total lipids into different classes, such as triacylglycerides (TAGs), diacylglycerides (DAGs), monoacylglycerides (MAGs), sterols, and steryl esters, and the level of each fraction was estimated.

We speculate that there is an inverse correlation between the abundance of FFAs and TAGs. The control strain (WT) demonstrated the maximum quantity in the TAG fraction (i.e., 32%) of the total lipid content (TLC) in comparison to the engineered strains (i.e., 18%, 22%, and 14% for M-1, M-2, and M-3, respectively). However, larger amounts of FFAs were detected in all engineered strains (i.e., 35%, 44%, and 31% for M-1, M-2, and M-3, respectively) in comparison to the control strain (1.2%).

Surprisingly, these non-native MCFAs were found to be integrated into all lipid classes. All fractions of total lipids for the control and engineered strains are depicted (Figure 8B). Our results demonstrated that all engineered strains contained significant quantities of MCFAs in the FFA and TAG fractions (Figure 8B).

In addition to the incorporation of MCFAs into the diverse classes of lipids, *TE* gene over-expression and sequential disruptions of *ACOX* and *ACOT* genes also influenced the localization of native fatty acids. In the context of C16 fatty acids, the mutant strains demonstrated elevated production of FFAs (i.e., 55%, 58%, and 40% of FFAs for M-1, M-2, and M-3, respectively) compared with 4.2% of FFAs in the control (Figure 8C). Concerning C18 fatty acid production, an incremental pattern of FFAs was also noticed in all mutant strains in comparison to the control strain (Figure 8D).

## 4. Discussion

### 4.1. The Impact of Heterologous Thioesterase (TE) Protein Over-Expression on Total Lipids and Fatty Acid Composition in the Mutant Fungal Strains

On a global scale, due to their natural ability to produce lipids in significant amounts, oleaginous microorganisms (i.e., Bacteria/yeast/fungi) are regarded as potential sources of different industrial commodities. Previously, several studies have described the potential of *M. circinelloides* to generate diverse compounds of biotechnological interest [9,14,15,63,64]. Like *Y. lipolytica* and other oleaginous microbes [20,37], *M. circinelloides* also produces LCFAs (i.e., C16–C18) in significant abundance. In addition, this fungus has no natural tendency to produce MCFAs due to the absence of MCFAs-producing pathways. In contrast, there is an increasing demand for MCFAs production as a substrate for the functional food and jet-fuel industries due to their unique physiochemical properties [6,18,35,36,37,38,39,40,41,42,43]. To achieve this goal, we first over-expressed the TE protein from *U. californica* in *M. circinelloides* strain MU758 to overproduce MCFAs. The resultant mutant (i.e., M-1) demonstrated a 1.42–1.63-fold increase in lipid production. One possible reason for the rise in total lipid accumulation in the aforesaid engineered strain can be attributed to particular function of TE protein in reducing the numerous feedback inhibition reactions (i.e., removing the inhibition of acyl-ACPs etc.) and consequentially, driving the elevated flux towards lipid production [37,65,66,67].

Interestingly, MCFAs production also increased up to 47.48%, with 10.41% and 6.35% increase for C8 and C10 fatty acids respectively, and 30.73% for C12. Concomitantly, the abundance of long chain fatty acids, (i.e., C16 and C18) declined from 0.28-fold to 0.47-fold. Heterologous TE protein localizes in the reaction center of fungal FASs to facilitate the access of acyl-ACP/acyl-CoA substrates to their catalytic sites. We observed that the increasing turnover of MCFAs to contribute the fatty acids abundance was directly correlated with the decline in the abundance of C16-containing and C18-containing lipids.This is possibly due to reason that acyl-ACP thioesterases having substrate specificities for medium-chain acyl-ACPs were incorporated into the native fungal FAS system and assisted the earlier discontinuation of fatty acid elongation to ultimately produce more quantities of MCFAs. This approach has been shown to serve as a potential tool to increase the carbon flux towards the fatty acid biosynthetic pathway (i.e., FASs) to achieve a significant abundance of MCFAs in *M. circinelloides.* Previous reports on *E. coli* and *Y. lipolytica* have described the low turnover of C8 and C10-containing lipids from the tested TE proteins [5,19,20,32,68,69,70].

Biologically, fatty acids are found in different classes of lipids, such as FFAs, sterols, steryl esters, TAGs, DAGs, and MAGs. This distribution allows the involvement of various enzymes with specific activity towards non-native fatty acids. Similar to our previous investigation, we noticed the presence of de novo fatty acids in all the aforesaid lipid fractions; therefore, characterization of the biosynthetic machinery (i.e., FASs) of *M. circinelloides* MU758 has the potential to allow the recognition and amendment of fatty acids as short as octanoic acid (i.e., C8). All mutant strains (i.e., M-1, M-2, and M-3) showed different quantities of various lipid classes. Generally, an antagonistic correlation was observed among TAGs, sterols, and FFAs proportions. Moreover, the quantity of FFAs increased in all mutant strains, with a corresponding reduction in the TAG and sterol proportions. Conversely, in the perspective of MCFAs, a similar trend was not observed, excluding a change in LCFAs length. The increased quantity of de novo MCFAs was associated with elevations in FFA abundance in all mutant strains.

### 4.2. Combined Impact of TEOver-Expression and ACOX (and ACOT) Gene Disruption on Lipid Accumulation and the Fatty Acid Portfolio in the Mutant Fungal Strains

For initial gene disruption step, we modified the β-oxidation pathway with the intention of further enhancing the MCFAs quantity. To achieve this goal, we first disrupted the peroxisomal *ACOX* gene responsible for the oxidation of MCFAs. We observed significant increase in total lipid accumulation in strain M-2 with lipid productivity of ~1800 mg/L.d and MCFAs productivity ~1100 mg/L.d, while the growth rate was negatively affected in this strain. Moreover, noteworthy increases (i.e., 26.63%) in the percentages of C8, C10, and C12-containing fatty acids were also observed in comparison to the M-1 strain. Peroxisomes of the engineered fungal strain from the current investigation (i.e., M-2) were genetically manipulated in such a controlled manner to degrade only long-chain fatty acids [24] and possible overproduction of medium-chain acyl-CoAs and acetyl-CoA. This aforesaid event indicating that degradation of LCFA can be an additional way of increasing MCFA different from that previously proposed [71]. The degraded acetyl-CoA product was converted into the active form called acetyl carnitine and then transported out of peroxisomes through carnitine acetyl-transferase shuttle system [58].In addition, transferase enzyme activity was present in *M. circinelloides* [4,7,19,20], which possibly facilitates the transportation of medium-chain acyl-CoAs and recycling of CoASH. Our results are consistent with a previous study of *S. cerevisiae* showing that genetic manipulation of peroxisomal *ACOX* genes led to increments in TLC and MCFAs production in the mutant strains [24]. Subsequent disruption of the specific *ACOT* gene in the M-3 strain drove stunted fungal growth with comparatively less lipid accumulation as well as reduced MCFAs (i.e., 37.59) and FFA production in comparison to M-1 strain. The M-3 strain eventually validated the involvement of the *ACOT* gene in the production of FFA and facilitated the transportation of MCFAs out of peroxisomes by specific membrane transporters [57]. Based on our above discussion, we assumed that the disruption of specific *ACOX* protein and presence of the *ACOT* gene in peroxisomes may facilitate the abundance of cytosolic medium-chain acyl-CoAs in the mutant strain (i.e., M-2), ultimately enhancing the production of FFAs and MCFAs. However, future studies are needed to estimate the concentration of medium-chain acyl-CoAs [24,72].

Furthermore, prior studies have also elaborated a modification in the β-oxidation pathway that switches the virulence potential of various fungal strains to a minimal level [55,73,74,75,76], but extensive investigations are needed to verify the outcome in this respect. Taken together, the current synergistic approach provided an innovative tool for MCFAs over-production in *M. circinelloides* strain MU758. However, outcomes related to the modification of the β-oxidation pathway demonstrated a persistent need for further investigations to oxidize more LCFAs and ultimately produce increased amounts of MCFAs. Our future investigations will focus on the genetic manipulation of β-oxidation pathway-related genes, such as other *ACOXs* and *ACOTs*, thiolase acetyl-synthetase [7,18,57], as well as the over-expression of diverse genes encoding lipase, acetyl-CoA carboxylase (ACC), and malic enzyme (ME), eventually to enhance the total fatty acid and, exclusively, the MCFAs contents [77,78,79,80].

## 5. Conclusions

In our current investigations, all mutant strains showed elevated production of total lipids (i.e., 1.25–1.47-fold) and MCFAs (i.e., 13–26-fold), especially the mutant strain M-2. Mutant strain M-2 due to synergistic effect of *TE* over-expression and β-oxidation pathway modification significantly augmented the MCFAs production. Finally, we also noticed the incorporation of MCFAs in all various classes of lipids. Hence, we concluded that *M. circinelloides* strain MU758 can be engineered to overproduce MCFAs with improved metabolic-flux through the FASs and β-oxidation pathways. The combined strategy of over-expressing TE protein and modifying the β-oxidation pathway provides a novel opportunity to produce diverse compounds of biotechnological interest.

## Figures and Tables

**Table 1:** List of strains and plasmid used in this investigation.

	Name	Genotype/Characteristics	References
*M. circinelloides*	MU758	*LeuA* ^-^ *pyrF* ^-^	This work
	MYS719	*LeuA* ^+^ *pyrF* ^-^ *TE +*	This work
	MYS720	*LeuA*^+^*pyrF*^+^*TE + acoxΔ* ::*pyrF*-*dpl237*	This work
	MYS721	*LeuA*^+^*pyrF*^+^*TE + acoxΔ*::*dpl*237	This work
	MYS722	*LeuA*^+^*pyrF*^+^*TE + acoxΔ*::*dpl*237	This work
	MSA321	*LeuA*^+^*pyrF*^+^*TE + acoxΔ*::*dpl*237 *acotΔ*::*pyrF*	This work
	MSA322	*LeuA*^+^*pyrF*^+^*TE + acoxΔ*::*dpl*237*acotΔ* ::*pyrF*	This work
Plasmids	pMAT1552-*LeuA*	Ampicillin^R^, *LeuA* Marker gene	(Zhang et al. [9])
	pMAT1552*-LeuA* TE	Contain *LeuA* Marker gene and thioesterase gene (*TE*)	This work
	pCR21-TOPO	Ampicillin ^R^ Kanamycin ^R^	Invitrogen
	pTOPO-pyrF	890-bp *pyrF* fragment cloned into pCR21-TOPO	This work
	pACOX-KO	*ACOX* disruption construct cloned into pCR21-TOPO	This work
	pACOT-KO	*ACOT* disruption construct cloned into pCR21-TOPO	This work
	pSL13	*pyrG* blaster marker in pCR21-TOPO	(Garcia et al. [49])
	pYS719	*PyrF* blaster marker in pCR21-TOPO	This work

**Table 2:** Fatty acid portfolio in control and mutant strains of *M. circinelloides* MU758.

	WT (Control)	M-1	M-2	M-3
**CDW (g/L)**	13.28 ± 1.37 ^a^	11.14 ± 0.21 ^b^	10.45 ± 1.26 ^b^	07.35 ± 1.29
**%TLC (CDW)**	36.70 ± 0.21	54.85 ± 1.20 ^b^	68.85 ± 2.20 ^a^	47.01 ± 2.40 ^c^
**TLC (g/L)**	04.87 ± 0.23	06.11 ± 0.12 ^a^	07.19 ± 0.08 ^a^	03.46 ± 0.09
**% MCFAs**	02.25 ± 1.10	47.45 ± 1.80 ^b^	60.09 ± 2.10 ^a^	29.61 ± 0.80 ^c^
**MCFAs (g/L)**	0.11 ± 0.71	02.88 ± 0.04 ^b^	04.32 ± 0.09 ^a^	01.04 ± 0.05
**P_TL_**	1217.5 ± 0.11 ^c^	1527.5 ± 0.41 ^b^	1800.5 ± 1.2 ^a^	865.1 ± 0.25
**P_MCFAs_**	204 ± 0.45	722 ± 0.23 ^b^	1084 ± 0.17 ^a^	260 ± 0.21

Fatty acid composition of the control (WT) and mutant strains (i.e., M-1, M-2, and M-3) of *M. circinelloides* (MU758). Total lipid (TLC) and MCFAs contents are provided in % w/w of CDW and % w/w of total lipid (g/L), respectively, for all strains. The MCFAs (C8–C12) contents are also presented in g/L. Lipid productivity (P_TL_) and MCFAs productivity (P_MCFAs_) were provided in mg/L.d. CDW, TLC and productivities (P_TL_, P_MCFAs_) were calculated after cultivation of the aforesaid strains over 72 h in modified K and R medium. The values represent the mean ± SD of three independent experiments. For different strains, with different genetic makeup, means with different letters were significantly different, with alphabetical order of letters (^a–c^) representative of the highest to lowest content. The values without a letter are considered statically non-significant.

## Supplementary Materials

The following are available online at https://www.mdpi.com/2073-4425/11/8/890/s1, Tables S1–S3. Primer used for this study. Figure S1. PCR amplification of genome of control (WT), and mutant strain (M-1). Figure S2. Confirmation of disruption of *ACOX* gene in M-1 mutant strain.

## Abbreviations

MCFAs	medium-chain fatty acids
LCFAs	long chain fatty acids
*TE*	Thioesterase
CDW	cell dry weight
M.C	*Mucor circinelloides*
FFA	free fatty acid
HR	homologous recombination
TLC	total lipid contents
ACP	acyl-carrier protein
FAME	fatty acid methyl esters
*ACOX*	acyl-CoA oxidase
*ACOT*	acyl-CoA thioesterase
FAS	fatty acid synthase
Vvm	volume of air per volume of medium per min
TAG	Triacylglycerol
DAG	Diacylglycerol
MAG	Monoacylglycerol
SE	steryl ester
MMC	minimal media with casamino acids
YPG	yeast peptone glucose
GC	gas chromatography
