# Supplementary material for: Increased Accumulation of Medium-Chain Fatty Acids by Dynamic Degradation of Long-Chain Fatty Acids in Mucor circinelloides
Source: Genes (Basel). 2020 Aug 5;11(8):890. doi: 10.3390/genes11080890 (PMC7464202; doi:10.3390/genes11080890)
Supplement: Supplementary file 1 [file genes-11-00890-s001.pdf]

Table S1. List of primers used in this investigation.

Name	5' → 3' Sequence	Remarks
YS-221	ACTTTTATATACAAAATAACTAAATCTCGAGATGGCTACCACCTCTTTGGC	Forward primer for *TE* gene over-expression
YS-222	ACTAGTCGCAATTGCCGCGGCTCGAGTTATTAGACACGAGGCTCA	Reverse primer for *TE* gene over-expression
YS-105	GAAGGCTTTACTGTTCCTATCA	Forward primer for integrated *TE* gene confirmation
YS-106	GACAGCAAGTGAGGAGAGGAACAGC	Reverse primer for integrated *TE* gene confirmation
YS-310	TTCTCATTCAGCAGCAGTGG	Primer for *ACOX* disruption, 5' region
YS-311	ACTGGCCGTCGTTTTACCCGTCAATGGTGAACATGAG	Primer for *ACOX* disruption, 5' region
YS-312	GTCATAGCTGTTTCCTGGGCTCCTGTTCTGCTAATGC	Primer for *ACOX* disruption, 3' region
YS-313	TCTTCTTTAAGCGCCACGTT	Primer for *ACOX* disruption, 3' region
YS-314	CTGCTATCAAGCGTCCCTTC	Nested forward primer for *ACOX* disruption
YS-315	CGAGAAATGGAGAGGCAAAG	Nested reverse primer for *ACOX* disruption
YS-351	TTCTCATTCAGCAGCAGTGG	5' outside forward of *ACOX* disruption confirmation
YS-352	TCTTCTTTAAGCGCCACGTT	3' outside reverse of *ACOX* disruption confirmation
YS-301	TACCGCTGTTGAACAAGCCA	Primer for *pyrF* to determine insertion
YS-302	TCATGGAGGAGAAGGGCACT	Primer for *pyr*F to determine insertion
AS-719	GTAAAACGACGGCCAGT	*5′ pyr*F blaster marker with 237-bp fragment
AS-720	CAGGAAACAGCTATGAC	*3′ pyr*F blaster marker with 237-bp fragment
YS-381	TGGTGAGAAAATCGCAGCCT	Forward primer for *ACOT* disruption
YS-382	GTTCAGACAGTGTGGGTGGT	Reverse primer for *ACOT* disruption
YS-353	GTAGTCAAAGCCGGCATCCT	5' forward of *ACOT* disruption confirmation
YS-354	AACAATGTCCAAGTTCGCGC	3' reverse of *ACOT* disruption confirmation

Table S2. List of primers for construction of *pyrF* blaster marker.

Name	5' → 3' Sequence	Remarks
YS-275	CTCGTTGCAGGATGACTCAA	5' forward Primer *pyrF* gene amplification
YS-276	TGCAACGTGCTACCCATTAG	3' forward Primer *pyrF* gene amplification
YS-277	TTCTCATTCAGCAGCAGTGG	5' 237-bp fragment with *XbaI* overhang
YS-278	ACTGGCCGTCGTTTTACCCGTCAATGGTGAACATGAG	3' 237-bp fragment with *NotI* overhang
YS-279	GTCATAGCTGTTTCCTGGGCTCCTGTTCTGCTAATGC	5' 237-bp fragment with *XbaI* overhang
YS-280	TCTTCTTTAAGCGCCACGTT	3' 237-bp fragment with *NotI* overhang
YS-281	CTGCTATCAAGCGTCCCTTC	Nested forward primer for overlapping PCR
YS-282	CGAGAAATGGAGAGGCAAAG	Nested reverse primer for overlapping PCR

Table S3. List of primers used in this investigation for qRT-PCR analysis.

Name	5' → 3' Sequence	Remarks
YS-412	GCTAAGTCTGTCGGTATCTTGG	Forward primer qRT-PCR analysis for *TE* gene over-expression
YS-413	ATACCGTTGTTACCAGAAGCAC	Reverse primer qRT-PCR analysis for *TE* gene over-expression
YS-414	GATTTGCAGGACCAAATTTCCAAGC	Forward primer RqT-PCR analysis for *ACOX*-KO
YS-415	CCCTCAGATCTCAGCAGATTAAACG	Reverse primer qRT-PCR analysis for *ACOX*-KO
YS-416	CAACCCACATTTGAAGCACGTCG	Forward primer qRT-PCR analysis for *ACOT*-KO
YS-417	CCACATGGAGTGATCCAAACTAGCC	Reverse primer qRT-PCR analysis for *ACOT*-KO

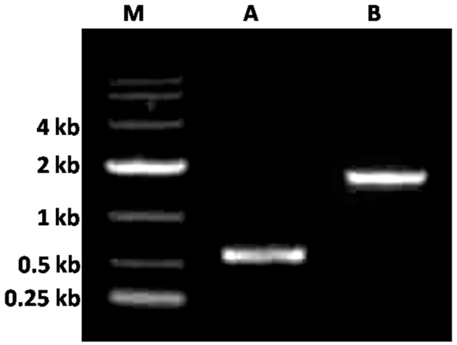

Figure S1. PCR amplification of genome of control (WT), and mutant strain (M-1) with the primers YS-105, and YS-106 (Table S1), represented as A, and B respectively. M, Gene Ruler DNA Ladder Mix. Sizes in kb of the relevant maker fragments are indicated.

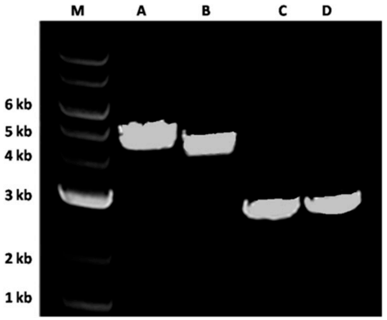

Figure S2. Confirmation of disruption of *ACOX* gene in M-1 mutant strain, M: demonstrated the Gene Ruler DNA Ladder Mix, A: MYS719 (LeuA$^-$ *pyrF*$^-$ TE+), B: MYS720 (LeuA$^-$ *pyrF*$^-$ *ACOX*Δ::*pyr*F-dpl237), C: MYS721 (LeuA$^-$ *pyrF*$^-$ *ACOX*Δ::*dpl*237), C: MYS722 (LeuA$^-$ *pyrF*$^-$ *ACOX*Δ::*dpl*237).
